# Identification and characterization of a novel major facilitator superfamily (MFS) efflux pump conferring multidrug resistance in *Staphylococcus aureus* and *Staphylococcus epidermidis*

**DOI:** 10.1128/aac.01739-24

**Published:** 2025-04-07

**Authors:** Honghao Huang, Yiyi Chen, Lingxuan Zhang, Peng Wan, Yan Chen, Yafei Li, Zhenling Zeng

**Affiliations:** 1 Guangdong Provincial Key Laboratory of Veterinary Pharmaceutics Development and Safety Evaluation, National Risk Assessment Laboratory for Antimicrobial Resistance of Animal Original Bacteria, College of Veterinary Medicine, South China Agricultural University, Guangzhou, China; 2 Department of Infectious Diseases, Sir Run Run Shaw Hospital, Zhejiang University School of Medicine, Hangzhou, Zhejiang, China; 3 Institute of Quality Standard and Monitoring Technology for Agro-products, Guangdong Academy of Agricultural Sciences 117866, Guangzhou, Guangdong, China; The Peter Doherty Institute for Infection and Immunity, Melbourne, Victoria, Australia

**Keywords:** *Staphylococcus aureus*, *Staphylococcus epidermidis*, major facilitator superfamily, efflux pump, antimicrobial resistance

## Abstract

A novel major facilitator superfamily (MFS) efflux pump in *Staphylococcus*, designated Nms, was identified via topology prediction. The secondary structure indicated the presence of 12 transmembrane segments (TMSs) and characteristic motif A of MFS efflux pumps. Experimental verification of efflux activity was conducted using ethidium bromide accumulation and efflux assays and biofilm formation assays. Antimicrobial susceptibility testing and efflux pump inhibition confirmed that Nms effectively effluxed various antimicrobial agents to confer multidrug resistance. Comprehensive genomic analyses were used to assess the prevalence and possible origins of the *nms* gene. The results revealed that the *nms* gene was present in *Staphylococcus aureus* ST398/ST541 and *Staphylococcus epidermidis* ST570/ST1166 strains from global isolates. The transmission of *nms* was associated with the prevalence of *S. aureus* ST398-t571 in swine-derived samples from China. Phylogenetic analysis revealed that *nms-*positive strains formed a distinct clade separate from other *S. aureus* ST398 strains. Genetic analysis of the *nms* gene revealed a significant presence of plasmid-related mobile genetic elements, with extended nucleotide sequences containing circular intermediates exhibiting high homology with those found in an *S. aureus* plasmid. These findings suggested that the *nms* gene likely initially originated from plasmids and subsequently integrated into chromosomes. In conclusion, Nms is a novel MFS efflux pump that confers multidrug resistance to *S. aureus* and has been carried predominantly by ST398-t571 isolates in recent years. Ongoing surveillance is essential to elucidate the origin of *nms* in *S. aureus*, particularly MRSA ST398-t571, and to understand the transmission among humans, animals, and the environment.

## INTRODUCTION

*Staphylococcus aureus* is a major Gram-positive pathogen that can cause serious infectious diseases, such as pneumonia, endocarditis, and bacteremia, in humans and animals (1). However, the emergence of antimicrobial resistance (AMR) in *S. aureus* has raised global public health concerns due to treatment failures (2). Efflux pumps play a significant role in the AMR of *S. aureus* by transporting various substrates, thereby reducing the intracellular concentration. The substrate profiles of these efflux pumps typically include antimicrobial agents and sanitizing compounds (3), leading to the development of multidrug resistance in *S. aureus* (4). Moreover, efflux activity may also be involved in the transport of extracellular polymeric substances (5, 6), quorum sensing and quenching molecules (7), all of which can impact and regulate biofilm matrix formation, thereby indirectly influencing biofilm development (8). In summary, the functional activity of efflux pumps is a critical research topic in understanding *S. aureus* and its resistance mechanisms.

In *S. aureus,* five families of efflux pumps have been identified, including multidrug and toxin extrusion (MATE), small multidrug resistance (SMR), major facilitator superfamily (MFS), ATP-binding cassette (ABC), and resistance nodulation division (RND). The MFS efflux pumps are typically composed of 12 or 14 transmembrane segments (TMSs) and highly conserved amino acid sequence motifs. Two kinds of motifs, motif A and motif C, are involved in the transporter function (9). Motif A of MFS is always located in the intracellular loop between the second and third TMSs. The TMS and motifs have also been used to identify novel MFS efflux pumps based on the secondary sequences of proteins (10). Notably, MFS efflux pumps have been confirmed to contribute to AMR in *S. aureus*, and the common substrates of MFS efflux pumps include tetracyclines (11), macrolides (12), quinolones (13), and amphenicols (14).

The MFS efflux pumps identified in *Staphylococcus* are encoded by various acquired antibiotic-resistant genes (ARGs) that can be transferred among *Staphylococcus* isolates, raising concerns about food safety and the spread of AMR (15, 16). These different MFS efflux pumps exhibit distinct dissemination patterns, with mobile genetic elements (MGEs), such as plasmids, genomic islands, and integrons, contributing to their prevalence within specific clonal lineages (17, 18). Therefore, investigating the prevalence of emerging MFS efflux pumps for early AMR control is essential.

A hypothetical efflux pump gene, *nms*, was identified in a retrospective study. Herein, the functional role of the *nms* was investigated through efflux activity assays, antimicrobial susceptibility testing, and genomic structural analysis.

## MATERIALS AND METHODS

### Strains and routine growth

To understand the origin and transmission of the *nms* gene, we scanned the global genomes through the GenBank public database and ultimately retrieved 42 *nms*-positive strain genomes including 37 *S*. *aureus* and 5 *Staphylococcus epidermidis* genomes (Table S1). There were 27 *S*. *aureus* isolates reported in previous studies by our research team (19, 20). To generate the phylogenetic tree of the *S. aureus* ST398 strains, 193 genomes were downloaded from the NCBI database (Table S2).

*Escherichia coli* DH5α was cultured in LB broth (Huankai, Guangzhou, China), and *S. aureus* strains were cultured in brain heart infusion (BHI) broth or agar (Huankai, Guangzhou, China). For plasmid maintenance, the medium was supplemented with antimicrobial agents at the following concentrations: 50 mg/L carbenicillin for *E. coli* and 20 mg/L chloramphenicol for *S. aureus*.

### Antimicrobial susceptibility testing

The minimum inhibitory concentrations (MICs) of antimicrobial agents were determined according to the guidelines of the Clinical and Laboratory Standards Institute (CLSI) (21, 22) using the broth microdilution method. Given that the tigecycline breakpoint was not included in the CLSI guidelines, it was defined in reference to the European Committee on Antimicrobial Susceptibility Testing (EUCAST)(23).

To evaluate efflux activity, the efflux pump inhibitor (EPI) carbonyl cyanide chlorophenylhydrazone (CCCP) was used at a final concentration of 100 µM for MIC determination.

### Gene cloning and electrotransformation

Gene cloning and electrotransformation were performed in a previous study (24). To generate the *nms*-carrying plasmid pLI50-Nms, the genomic DNA of *nms*-positive strain GD4SA108 was extracted using a commercial kit (Tiangen, Beijing, China), and the *nms* gene and upstream 320 bp region as a possible promoter region were amplified using the primers F1 (5′- CCGGGTACCGAGCTCGAATTCGATACACCTTTTTATATCG-3′) and R1 (5′- GGCCCTTTCGTCTTCAAGAATTCGCATGAAACTAATCACTTGA-3′) and subsequently cloned and inserted into the shuttle vector pLI50 (25), which was linearized with *Eco*RI (NEB, Ipswich, USA) using an infusion kit (Tsingke, Beijing, China).

The *S. aureus* strain RN4220 does not carry Qac, Mmr, or another efflux pump that may impact the functional experiment of Nms and was selected as the host to acquire electrotransformants. *S. aureus* RN4220 competent cells were prepared with 0.5 M aqueous sucrose, and electroporation was performed with the MicroPulser electroporator (Bio-Rad, Hercules, USA). PCR amplification and Sanger sequencing were performed using the primers F2 (5′-AGGTGTCATAGGTGGAT-3′) and R2 (5′-TTTCTTTGTAGGTGCTT-3′) to screen and confirm *nms*-positive electrotransformants. The plasmids and strains used in this study are listed in Table S3.

### Intracellular accumulation and efflux activity assays

The overnight-grown strains were incubated (dilution 1/100) in fresh broth at 37°C and 180 rpm for over 6 h, after which they were centrifuged and resuspended twice in phosphate-buffered saline (PBS). PBS supplemented with 10 mM glucose was used for resuspension, and the OD600 was adjusted to approximately 0.6. For the intracellular accumulation assay, ethidium bromide (EtBr) was added to 200 µL of each cell suspension to a final concentration of 2 mg/L, and the fluorescence intensity was measured every 10 min at emission and excitation wavelengths of 580 and 500 nm, respectively. For the efflux assay, EtBr was added to the suspensions of cells at a final concentration of 2 mg/L at room temperature for 1 h. Then, the suspensions were centrifuged and resuspended twice in PBS to remove the extracellular EtBr, and the fluorescence intensity was measured. The measurements were the same as those of the accumulation assay. To execute the efflux inhibition, CCCP was added at a final concentration of 100 µM after 20 min.

### Biofilm formation assays

The overnight-grown strains were diluted 1:200 into 200 µL of trypticase soy broth supplemented with 0.5% glucose in a 96-well plate and incubated at 37°C for 24 h. After incubation, the remaining broth was carefully removed, and the wells were washed three times with 200 µL of PBS. Subsequently, 100 µL of 0.1% crystal violet was added to each well and allowed to stain for 5 min at room temperature. The wells were then washed again three times with 200 µL of PBS. The stained biofilms were then dried at 60°C, after which 200 µL of acidified ethanol was added to each well to suspend the stained biofilm. The resulting suspension was then transferred to a fresh 96-well plate, and the OD600 was measured immediately.

### Bioinformatics analysis

The molecular types were identified using mlst (https://github.com/tseemann/mlst) and spaTyper (https://github.com/mjsull/spa_typing). The virulence factors (VFs) and ARGs were identified using ABRicate v1.0.1 (https://github.com/tseemann/abricate) based on supported databases updated in August 2024. To scan the global genomic data, a unique database was generated in ABRicate v1.0.1, and the nucleotide sequences of the *nms* gene were detected using blastn (https://github.com/JacobLondon/Blastn). The genomic data of *nms*-positive *Staphylococcus* spp. strains were downloaded from the GenBank database (Table S1). The core–genome alignment of *nms*-positive *S. aureus* strains were constructed by Parsnp (26) with a random reference. A phylogenetic tree of *nms*-positive *S. aureus* strains was generated with a GTRGAMMA replacement model based on maximum likelihood (ML) using RaxML (27). The WGS data of the strains were annotated using Prokka v1.14.6 (28). The linear maps of the genomic comparisons were inferred using Easyfig v2.2.5 (29). The genomic islands were predicted in IslandViewer 4 (30).

The TMS of the Nms efflux pump was predicted using TMHMM 2.0 (https://services.healthtech.dtu.dk/services/TMHMM-2.0/). SWISS-MODEL (https://swissmodel.expasy.org/) was used to predict the 3D structure of the protein. Protter v1.0 was used to visualize the topology of the Nms efflux pump (https://wlab.ethz.ch/protter/start/). MEGA-X (31) was used to align the amino acid sequences of the MFS efflux pumps including the *nms* with preset ClustalX and generate a phylogenetic tree based on the neighbor-joining (NJ) algorithm.

## RESULTS

### Identification of a novel MFS efflux pump gene in *S. aureus*

**Fig 1:**
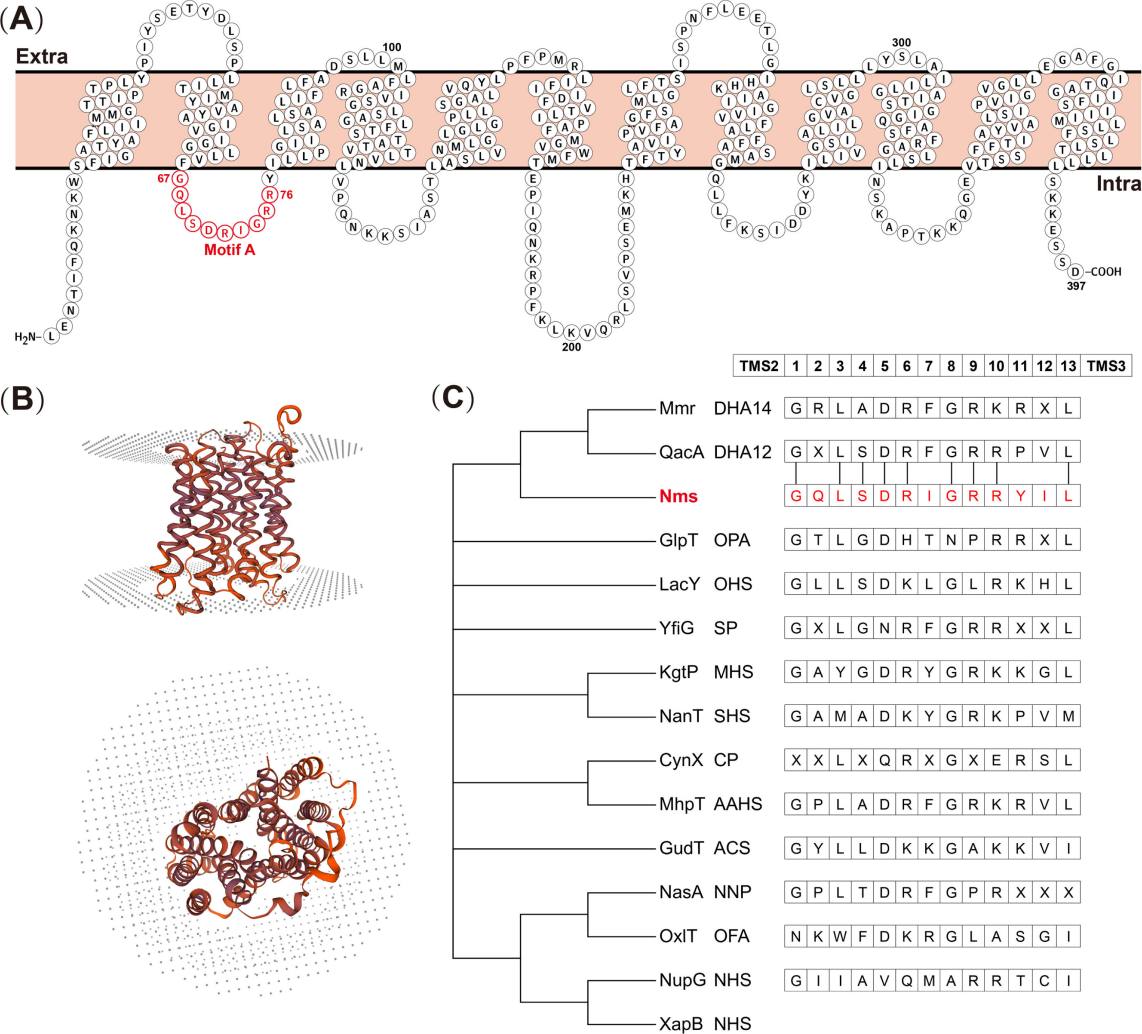
Topology and phylogenetic analysis of a novel MFS efflux pump in *S. aureus*. (**A**) The transmembrane topology of Nms protein was generated by Protter. The circles presented the amino acid sequences, and sequences of motif A were highlighted in red. (**B**) The phylogenetic tree was generated by MEGA-X based on amino acid alignment using ClustalW. The names of efflux pumps were presented together with groups, and Nms was highlighted in red. (**C**) The phylogenetic tree of Nms and the other known MFS. The predominant residues between TMS2 and TMS3 of different groups were listed. The connecting cables denote similar residues between Nms and the DHA12 group.

In the genomes of *S. aureus* isolates, a gene with a consistent motif of the MFS, which contains the amino acids G(X)3DK/RXGRR/K and is recognized as motif A, was identified. This gene was annotated as a putative transporter protein in the previous genome. The gene has a length of 1,194 bp and was calculated to encode a 42.8 kDa protein. The prediction of gene products revealed that the amino acids are arranged as 12 TMSs, which is the foundational structure of MFS transporters (Fig. S1). Further topology analysis demonstrated that motif A is an intramembrane loop between TMS2 and TMS3 (Fig. 1A). The evidence described above was sufficiently compelling to identify a novel MFS transporter in *S. aureus*. For advanced research, we named the gene and product *nms* (Novel MFS in *Staphylococcus*) and Nms, respectively. Phylogenetic analysis of the identified efflux pumps in the MFS groups and Nms revealed that Nms was on the branch that included 12-TMS drug:H^+^ antiporter (DHA12) and 14-TMS drug:H^+^ antiporter (DHA14) (Fig. 1C). An alignment of amino acid residues among Nms, QacA, and Mmr, which were clustered on a branch, was performed (Fig. S2). Compared with Nms, QacA exhibited 20.23% identity and 46.24% similarity, and Mmr exhibited 22.51% identity and 40.54% similarity.

### Verification of the efflux activity of NMS

To evaluate the efflux activity of the Nms efflux pump, EtBr accumulation, EtBr efflux, and biofilm formation assays were performed on *S. aureus* strains RN4220 (blank), RN4220-pLI50 (*nms*-negative), and RN4220-pLI50-Nms (*nms*-positive).

**Fig 2:**
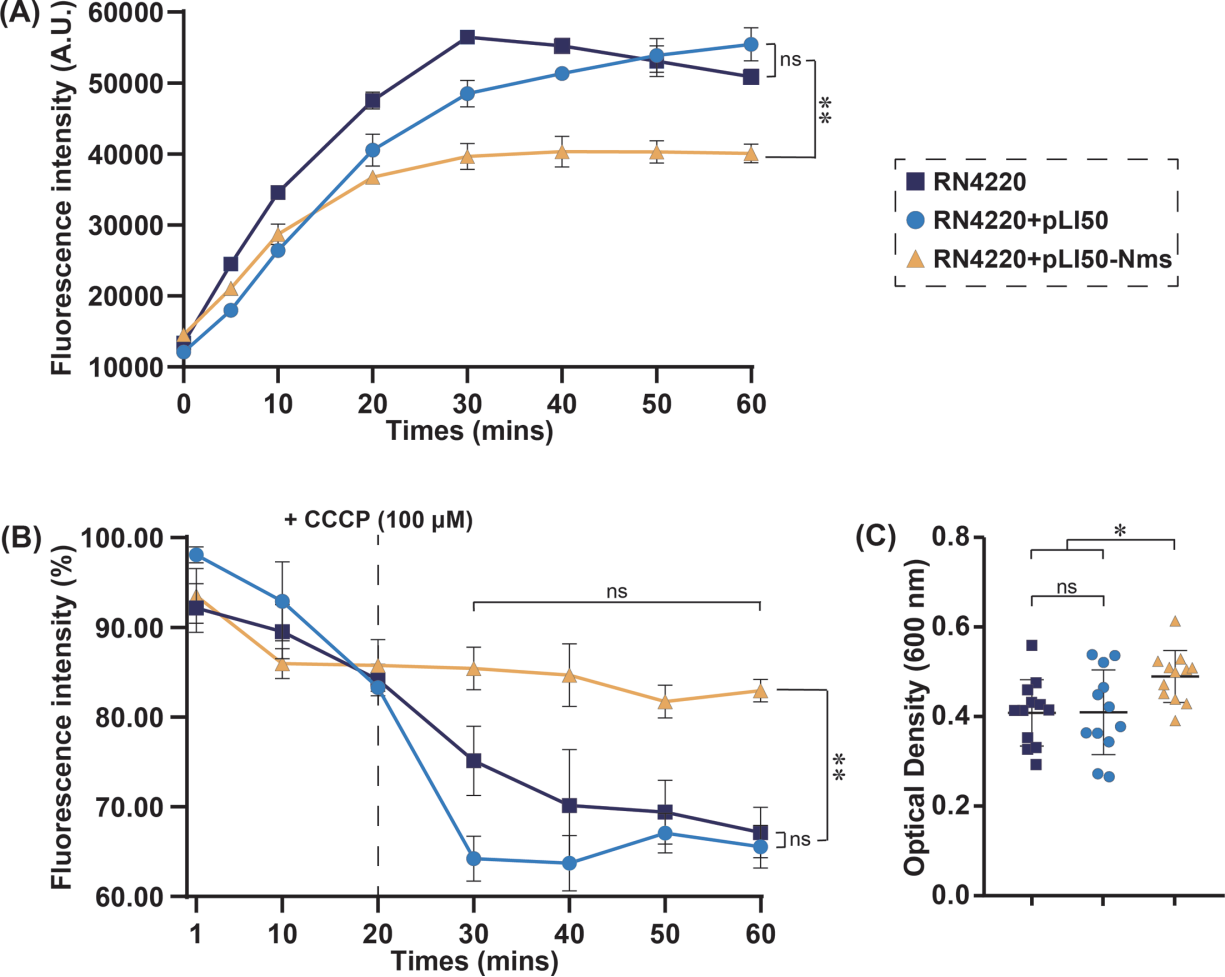
Verification of efflux activity of Nms in *nms*-positive *S. aureus* transformant. (**A**) EtBr intracellular accumulation assays. Three groups of *S. aureus* strains were adjusted to the same optical density and treated with EtBr; fluorescence intensities were determined every 10 min for 1 h. (**B**) EtBr efflux assays. Three groups of *S. aureus* strains were treated with EtBr for 1 h and moved by PBS, then measurements were performed every 10 min for 1 h. The data of time points were estimated using percentages of the fluorescence intensity of initial fluorescence intensity. (**C**) The biofilm formation assays. Three groups of *S. aureus* strains grown in TSB broth supplemented with glucose and biofilms were stained by crystal violet. The biofilm formation was determined by OD600. All figures used the same legend. All data were presented as the mean of three independent tests, and the paired *t*-tests were performed to analyze data. ns, no significance; *, *P* ≤ 0.05; **, *P* ≤ 0.01.

The results of the accumulation assays revealed that the fluorescence intensities of the *nms*-positive strains were lower than those of blank and *nms-*negative strains after 20 min and were statistically significantly lower throughout the entire measurement period (Fig. 2A).

The results of the efflux activity assays revealed that the efflux ratios were similar before the addition of CCCP. After the addition of CCCP, the efflux activities of the *nms*-positive strains were inhibited, and their fluorescence intensities were not significantly different; in contrast, those of the blank and *nms-*negative strains consistently decreased. Overall, the fluorescence intensities of the *nms*-positive strains were significantly greater than those of other strains (Fig. 2B).

There was no significant difference in biofilm formation between the blank and *nms-*negative strains in the biofilm formation assays, but *nms*-positive strains presented statistically significant increases in biofilm formation, approximately 20% greater than those of other strains (Fig. 2C).

### Nms confers multidrug resistance in *S. aureus*

**Fig 3:**
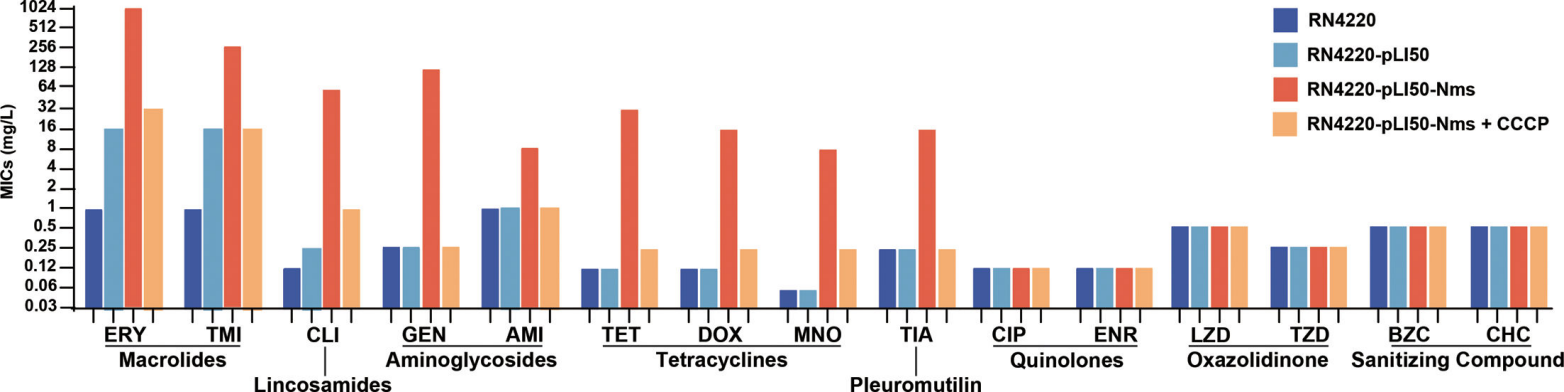
The MICs and efflux pump inhibition of nms-positive transformants. Abbreviation: ERY, erythromycin; TMI, tilmicosin; CLI, clindamycin; GEN, gentamicin; AMK, amikacin; TET, tetracycline; DOX, doxycycline; MNO, minocycline; TGC, tigecycline; TIA, tiamulin; CIP, ciprofloxacin; ENR, enrofloxacin; LZD, linezolid; TZD, torezolid; BZC, benzalkonium chloride; CHC, chlorhexidine hydrochloride. *Staphylococcus aureus* ATCC 29213 was set as the quality control strain. To perform the efflux pump inhibition, the MICs are determined repeatedly in MH broth supplemented with carbonyl cyanide 3-chlorophenylhydrazone (CCCP, 100 µM).

The susceptibilities of various antimicrobial agents, including erythromycin, tilmicosin, clindamycin, gentamicin, amikacin, tetracycline, doxycycline, minocycline, and tiamulin, were altered in different testing groups (Fig. 3). The MICs of strain RN4220-pLI50-Nms against nine drugs of five classes increased by 4- to 256-fold (0.06–16 to 0.12–1024 mg/L) compared with those of RN4220-pLI50. The MICs of RN4220-pLI50-Nms broke the resistance breakpoints of eight drugs and the intermediate breakpoint of minocycline with an increase of 128-fold (0.06 to 8 mg/L). However, the susceptibilities of tigecycline, quinolones, oxazolidinones, and sanitizing compounds were not affected (i.e., ≤2-fold changes). With the addition of EPI CCCP, the MICs of antimicrobial agents that were impacted by Nms were restored below the susceptible breakpoints, except erythromycin. In terms of increasing folds, the efflux activities of strains encoding Nms were greater than those of other strains against lincosamides and tetracyclines.

### Global prevalence of *nms*-positive *S. aureus*

**Fig 4:**
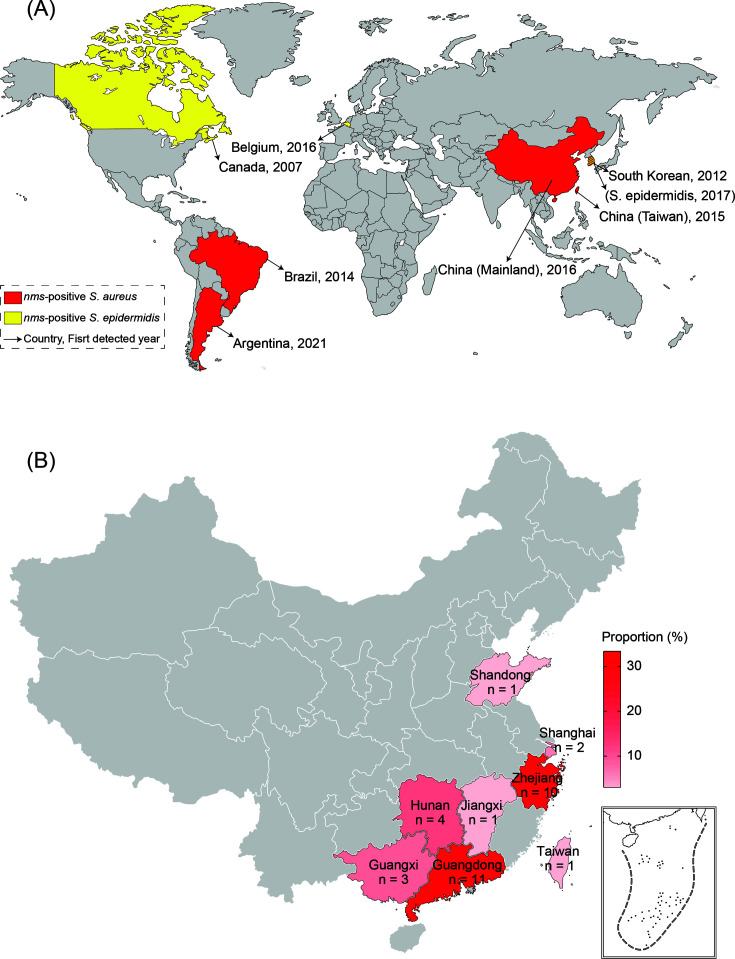
Global prevalence of *nms*-positive *S. aureus*. The *nms* gene was detected in various countries in 2007–2021. (**A**) The countries detected with *nms*-positive *S. aureus* were highlighted in red, and those detected with *nms*-positive *S. epidermidis* were highlighted in yellow. The arrows pointed to the names of the countries and the years of the first report. (**B**) The majority of *nms*-positive *S. aureus* strains were isolated in China, and a more detailed map down to the provinces indicated that the isolates were reported in eastern and southern China. The depth of the red represents the reported proportion of the province in China. Source: Standard Map Service website of the Ministry of Natural Resources of China.

To understand the current prevalence of *nms*, 37 genomes of *S. aureus*, including 27 isolates from previous studies by our research team, were selected by screening the *nms* gene. Analysis of the isolates revealed that the *nms* gene in *S. aureus* was detected in a swine-origin sample from South Korea in 2012 at the earliest and was consistently prevalent from 2012 to 2021 in four countries on two continents (Fig. 4A). Most strains were isolated in China (86.49%, 32/37) and spread across eight provinces in the eastern and southern areas (Fig. 4B). The most prevalent strains were ST398 (94.59%, 35/37), and 64.86% (24/37) were identified as *spa* type t571. Almost all strains were isolated from swine and swine-relevant environments, in addition to an isolate from Taiwan, China, in 2019 and an isolate from South Korea in 2017.

**Fig 5:**
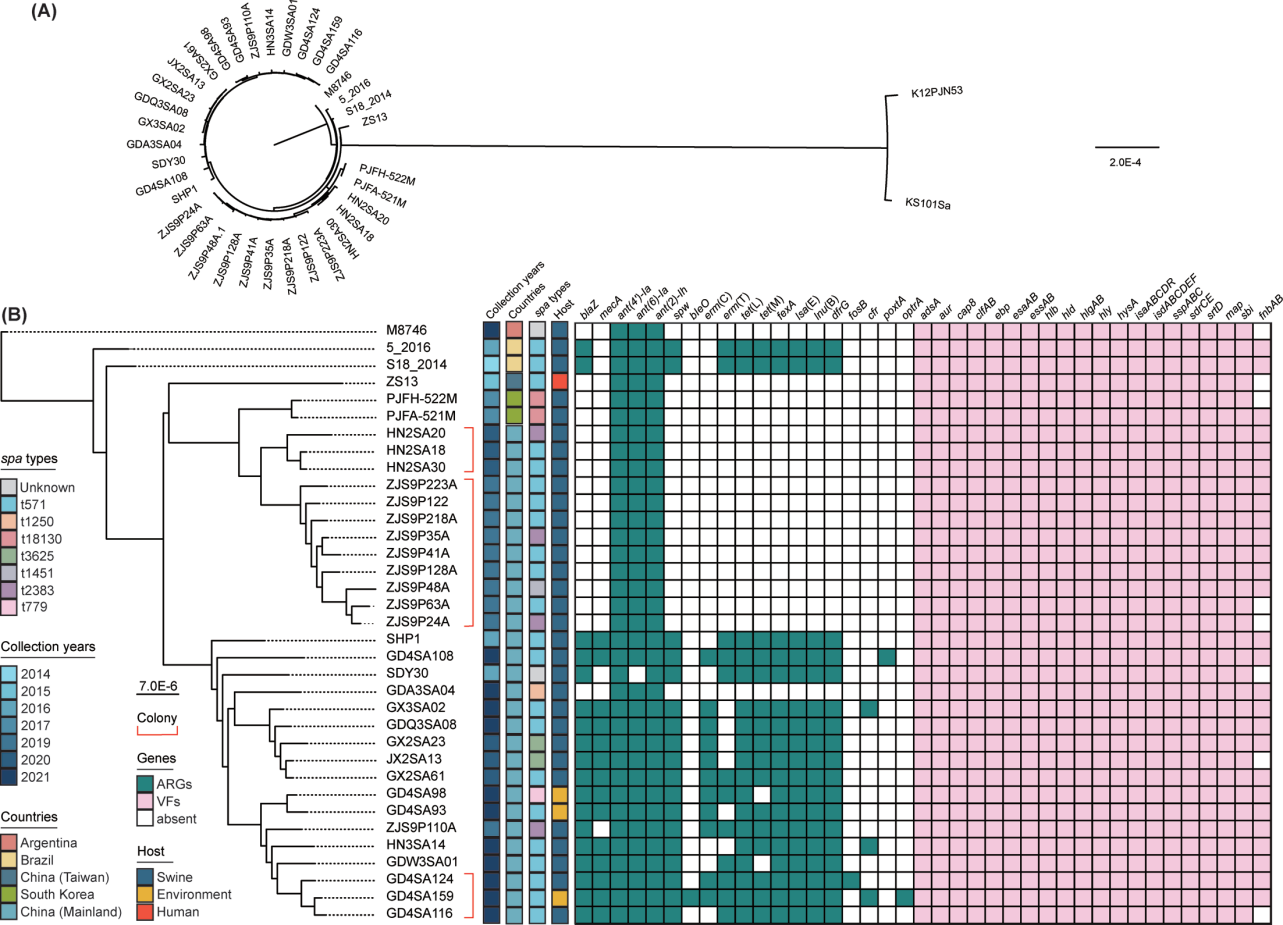
Phylogenetic analysis of *nms*-positive *S. aureus*. (**A**) Phylogenetic tree of all retrieved *nms*-positive *S. aureus* isolates. The *S. aureus* ST541 isolates K12PJN53 and KS101Sa were separated from the others. (**B**) Phylogenetic tree of *nms-*positive *S. aureus* ST398 isolates. The information was listed behind the tree, including the countries, collection years, spa types, antibiotic resistance genes (ARGs), and virulence factors (VFs) abundance map.

Phylogenetic analysis revealed that the *S. aureus* ST398 strains were dissimilar to the *S. aureus* ST541 strains that were previously isolated (Fig. 5B); therefore, a detailed phylogenetic tree of all 35 *S*. *aureus* ST398 strains was constructed (Fig. 5C). Based on the isolation location and time, three colony groups of 15 strains were defined. The *nms*-positive *S. aureus* ST398 strains carried mostly 17 kinds of ARGs that could confer resistance to β-lactams, aminoglycosides, bleomycin, macrolides, tetracyclines, amphenicols, lincosamides, fosfomycin, and oxazolidinones. VFs related to the colonization and infection of *S. aureus* were detected in all strains.

### Genetic contents of *nms* and possible origin

To elucidate the origin of the *nms*, we expanded the scanning genomic range to include other *Staphylococcus* spp. strains. Five *S*. *epidermidis* strains carrying the *nms* gene were identified (Table S1). The *nms* gene first appeared in *S. epidermidis* isolated from cows in Canada in 2007. The *nms-*positive *S. epidermidis* strains were identified as ST570 (3/5) and ST1166 (2/5).

**Fig 6:**
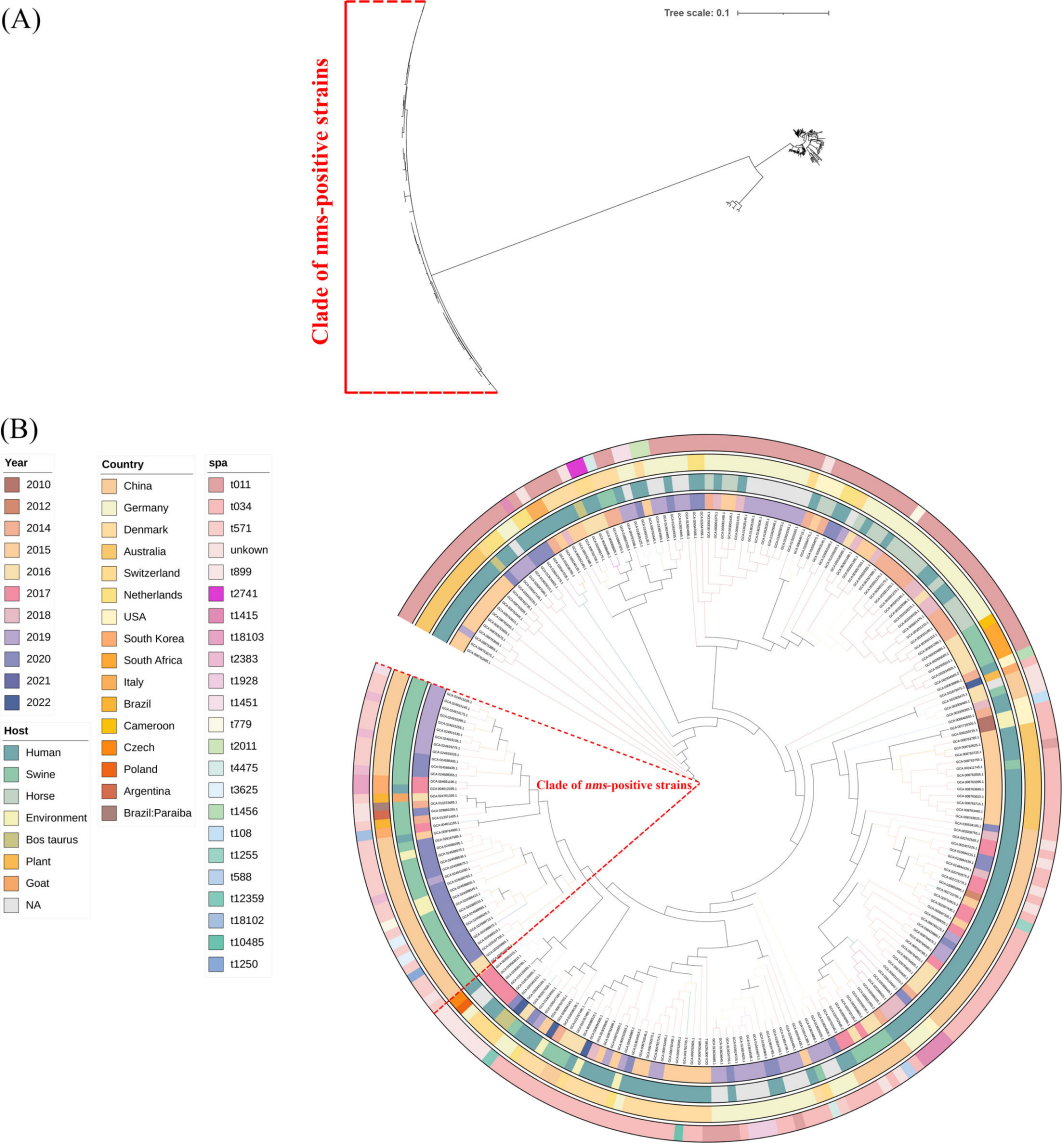
Phylogenetic analysis of *S. aureus* ST398 reported in the last 10 years including *nms*-positive strains. A total of 230 genomes of *S. aureus* ST398 strains were used to generate a phylogenetic tree. (**A**) The phylogenetic tree of *S. aureus* ST398 strains with distances. A clade of *nms-*positive strains was isolated from the other strains and marked with red lines. (**B**) The phylogenetic tree with labels and rectangles of detailed information listed around the tree.

As mentioned above, *S. aureus* ST398 strains play an important role in the prevalence of *nms*-positive strains. To clarify the evolutionary relationships of *nms*-positive *S. aureus* ST398 and the other strains, a total of 193 genomes of *S. aureus* ST398 isolated in the last 10 years were identified and downloaded from the NCBI database. The *nms*-positive *S. aureus* strains were separated and located in a clade (Fig. 6A), and only two strains from South Korea (GCA_004011165.1) and Brazil (GCA_013372685.1) were clustered with them in the tree (Fig. 6B).

**Fig 7:**
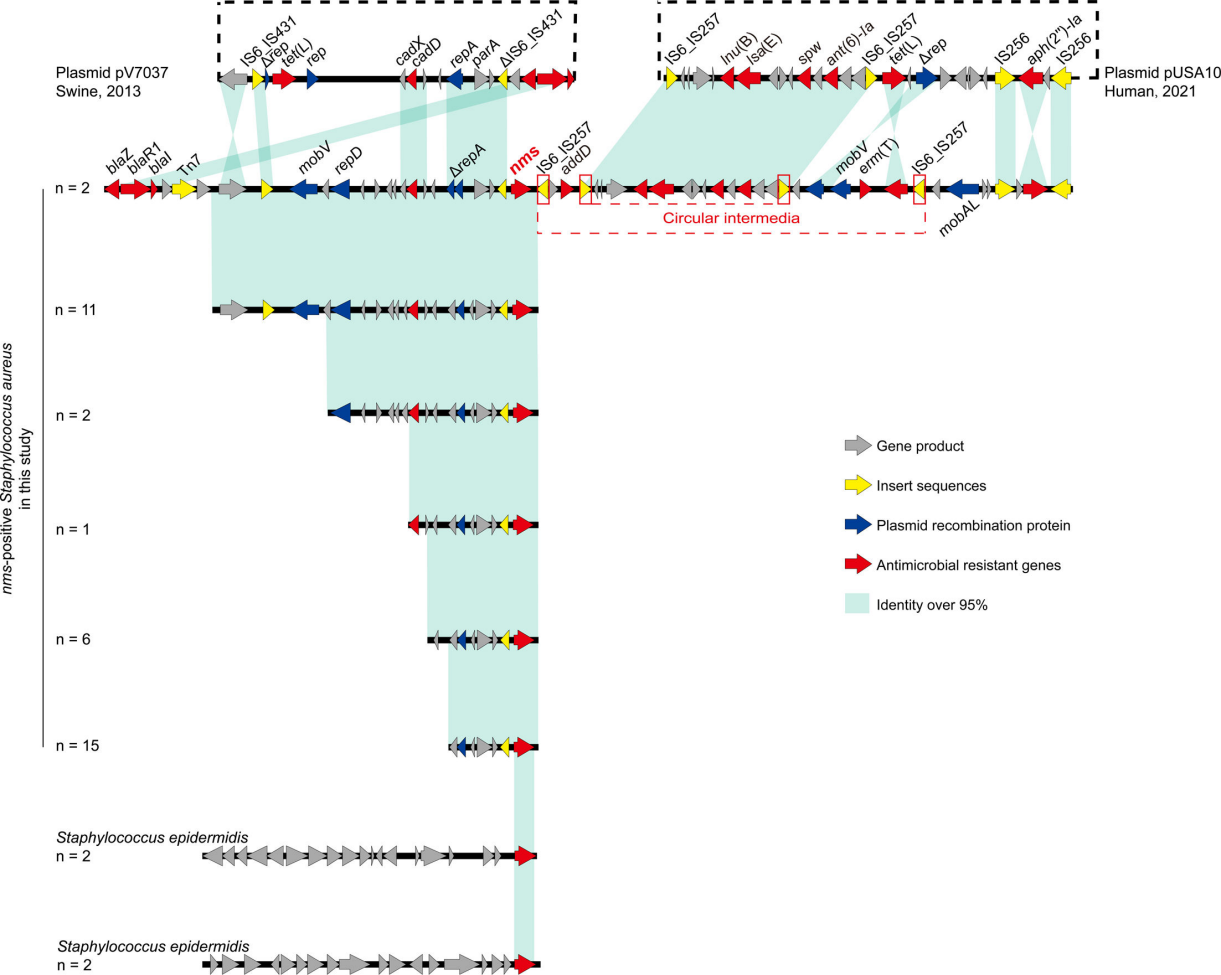
Genetic comparison linear map of the *Nms* gene. The genetic content of *nms* from *nms-*positive strains isolated in different sources and times was compared. Arrows were used to present gene products, insert sequences (IS), plasmid recombination proteins, and antibiotic resistance genes (ARGs) were highlighted with colors as the legends showed.

The genetic contents of *nms*-positive strains isolated from different sources and countries were compared and visualized on a linear map (Fig. 7). The *nms*-positive strains presented highly similar genetic contents. In the chromosomes of *S. aureus* GD4SA108 and GD4SA159, various ARGs and MGEs were detected upstream and downstream and identified in integrative genomic islands with *nms* (Table S4). In the genomic islands, the IS*6* family frequently occurred with *nms* and mediated the circular intermediates containing *addD, lnu*(B), *lsa*(E), *spw*, and *ant(6)-Ia*. The plasmid-associated MGEs were detected in the chromosomal genetic contents of *nms* and were similar to the plasmid pV7037 from swine-origin *S. aureus* SA7037 (GenBank: HF586889.1) and plasmid pUSA10 from prevalent human *S. aureus* strains (GenBank: CP165633.1). Notably, the genetic contents of *nms* in *S. epidermidis* were similar to those in *S. aureus* upstream or downstream.

## DISCUSSION

To date in August 2024, 180 records of MFS have been presented in the reference catalog of efflux pumps in the NCBI database. However, a previous *in silico* study revealed that the multidrug efflux pump genes contained in the genome of *S. aureus* have not yet been identified (32). Given that efflux pumps play important roles in *S. aureus* infection and treatment, it is necessary to identify novel MFS efflux pumps and explore the prevalent risks worldwide.

The typical 12 TMS and motif A were detected in Nms, providing compelling evidence that it functions as an MFS efflux pump. To our knowledge, the MFS efflux pumps are currently classified into 17 groups based on amino acid sequence similarities, and the occurrence of these pumps in bacteria has been confirmed in 15 groups (33). For a nuanced understanding, we chose one representative efflux pump per group and one more nucleoside:H^+^ symporter (NHS) efflux pump as the control for phylogenetic analysis; the selected representative efflux pumps were reported in *Staphylococcus* or *Escherichia* strains (33). Two NHS efflux pumps were aggregated into a branch in the generated phylogenetic tree, and Nms was in the same branch including the DHA12 and DHA14 groups, which differ in their number of TMSs. Motif A of the MFS was consistently located between TMS2 and TMS3, where the amino acid residues exhibited polymorphisms across different groups. The comparison revealed that motif A of Nms aligned similarly with predominant residues between TMS2 and TMS3 of the DHA12 group (GXLSDRFGRRPVL) (33). Overall, Nms was inferred to be a member of the DHA12 group, MFS, according to the number of TMSs and amino acid residues of motif A.

To perform the functional verification experiments, the single-copy shuttle vector pLI50 was chosen given that the *nms* gene was positioned in the chromosome. EtBr is a classic substrate of bacterial efflux pumps used to confirm the potency of efflux activity (34). CCCP is a standard EPI that inhibits H^+^-independent efflux pumps in *S. aureus* (35). In EtBr accumulation assays, the presence of *nms* consistently decreased the fluorescence intensities caused by the uptake of EtBr. In the efflux assays, the addition of CCCP disrupted the rapid decrease in fluorescence intensity caused by the efflux activity of Nms. The tendencies and efficiencies of the EtBr assays were similar to those of the previous identification of another novel MFS efflux pump (36).

The promotion of biofilm formation is associated with efflux activity. Efflux pump genes, such as *norB*, *norC*, and *medA*, were found to be overexpressed in biofilm-forming *S. aureus* cells, with expression levels increasing by 4.6 to 7.0 times compared with those in planktonic colonies (37). Interestingly, to date, only the MFS has been confirmed to have a direct impact on *S. aureus* biofilm formation (38). In this study, the *nms*-positive transformant strain also demonstrated enhanced biofilm formation. Increased biofilm formation was associated with a greater risk of AMR development (39) and created facilitating conditions to allow the transfer of ARGs (40).

To evaluate the effect of efflux activity against antimicrobial agents, the MICs of the groups were determined. The agents that decreased susceptibilities included routine treatment options in humans and veterinary medicine, such as macrolides, lincosamides, aminoglycosides, tetracyclines, and pleuromutilin. The restored susceptibility with the addition of EPIs indicated that efflux activity contributed to AMR. It should be explained that the vector pLI50 carried the chloramphenicol-resistant gene for screening, decreasing macrolide susceptibility simultaneously, but pLI50-Nms conferred higher-level resistance against erythromycin, which was still sufficient enough to infer that Nms contributed to the macrolide resistance. Although there was a close relationship between Nms and QacA, no impact of sanitizing compounds was detected in RN4220-pLI50-Nms. The identified MFS efflux pumps in *S. aureus* confer multidrug resistance; for example, the substrates of NorA include fluoroquinolones, dyes, antiseptics, and quaternary ammonium compounds (41), and LmrS contributes to the efflux of lincosamides and linezolid (42). These cases, including Nms, underscore the urgency of identifying and reporting MFS efflux pumps for clinical reference. Compared with NorA and LmrS, Nms seemed to confer higher folds of antimicrobial resistance. However, this evaluation was not fully quantifiable given that different plasmids do not exhibit recurrent expression in the chromosome of *S. aureus*.

A screen of global genomes revealed that 37 *S. aureus* strains and 5 *S*. *epidermidis* strains harbored the *nms* gene, and ST398 emerged as the dominant type among the *nms*-positive *S. aureus* isolates. Previous genomic research has indicated that *S. aureus* ST398 strains pose risks of clonal expansion in humans, animals, and the environment (43, 44). Over the past few decades, *S. aureus* ST398 isolates have predominated in swine and humans in Europe and the USA (45) but have been sporadically identified in China (46). However, recent reports have shown that *S. aureus* ST398 isolates are now predominant in swine-derived samples in China (47, 48). Notably, *S. aureus* ST398 strains exhibit high virulence and biofilm formation capacity (49), whereas *S. aureus* isolates identified as t571 are relevant to infection and colonization (50, 51). These two types are frequently identified together, with *S. aureus* ST398-t571 recognized as a potential threat to both humans and animals (52). In this study, we found that ST398-t571 was the dominant type (64.86%, 24/37) among the *nms*-positive *S. aureus* isolates, with methicillin-resistant *S. aureus* (MRSA) strains constituting more than half (58.33%, 14/24) of these lineages. These findings were the same as those of earlier studies reporting that MRSA ST398-t571 strains carry more abundant ARGs and VFs than methicillin-susceptible *S. aureus* (MSSA) strains (19, 20). The *nms*-positive MRSA ST398-t571 strains (PJFA-521M and PJFH-522M) were isolated from a farmer and his pigs, demonstrating the zoonotic spillover potential of *nms* carried by MRSA ST398-t571.

Phylogenetic reconstruction revealed monophyletic clustering of *nms-*positive strains among 192 strains of *S. aureus* ST398, with two sporadic *nms*-negative isolates from South Korea and Brazil, where the earliest *nms-*positive *S. aureus* strains have been reported. This suggests horizontal acquisition of *nms* by *S. aureus* ST398 strains in these regions. *S. epidermidis* is a coagulase-negative *Staphylococcus* (CoNS) and serves as a reservoir of ARGs for *S. aureus* (53). The *nms* gene was detected in *S. epidermidis* before it was detected in *S. aureus. S. epidermidis* ST570 strains have frequently been reported among swine-origin CoNS strains and carry abundant ARGs (54). Nonetheless, *S. epidermidis* strains should not be ignored as potential sources of the *nms* gene, given that their genetic contents differ significantly.

The *nms* gene is embedded within a genomic island flanked by IS6 elements (IS*257* and IS*431*), known mediators of plasmid-chromosome integration in *S. aureus* (55), mirroring documented IS-driven recombination events in *tet*(L)- and *cadDX*-positive plasmids (56, 57). Interestingly, both examples were documented in *S. aureus* ST398 strains. The co-localization of intact *repD* and truncated Δ*repA* with the plasmid relaxase genes *mobA*/*mobV* (58) provides mechanistic evidence for plasmid-to-chromosome transmission in ST398 lineages. In *nms-*positive *S. aureus* GD4SA108 and GD4SA159, four IS*257* elements were identified as the molecular basis for two circular intermediates that facilitate the transmission of ARGs. The IS*257*-derived hypothetical circular intermediates were also detected in the *S. aureus* plasmid pUSA10 and showed high homology with the genetic contents of *nms*. Based on current evidence, we hypothesize that the *nms* gene originated in plasmids and was transmitted through various lineages of *S. aureus*, notably ST541 and ST398, with plasmid integration into chromosomes, whereas the *S. aureus* ST398 strains promoted the spread of *nms* by clonal expansion.

### Conclusion

The *nms* gene encodes a novel MFS efflux pump, Nms, which is classified into the DHA12 group. The substrates of Nms include EtBr and five classes of antimicrobial agents and can enhance biofilm formation. To date, the *nms* gene is prevalent worldwide and is carried mainly by *S. aureus* ST398 strains. The *nms*-positive MRSA ST398-t571 strains should be recognized as an emerging public health threat causing increasing public health concerns. The *nms*-positive strains were highly correlated with livestock, particularly swine. The *nms* gene likely originated from plasmids and subsequently integrated into chromosomes, and IS*6* family MGEs have played important roles in its transmission. *S. epidermidis* should be considered a potential origin of the *nms* gene. It is necessary to survey the prevalence of *nms*-positive *S. aureus* and identify credible evidence to understand its origin and dissemination.

## SUPPLEMENTAL MATERIAL

Fig. S1 aac.01739-24-s0001.jpg Foundational structure predicted by TMHMM 2.0.

Fig. S2 aac.01739-24-s0002.jpg Alignment of amino acid residues of QacA, Nms, and Mmr.

Table S1 aac.01739-24-s0003.xlsx Genomic information of *nms*-positive *S. aureus* and *S. epidermidis* strains.

Table S2 aac.01739-24-s0004.xlsx Genomic information of *S. aureus* ST398 reported in the last 10 years, including *nms*-positive strains.

Table S3 aac.01739-24-s0005.docx Strains and plasmids used in the study.

Table S4 aac.01739-24-s0006.xlsx Genomic islands in GD4A108 and GD4A159 predicted by IslandViewer 4.

ASM does not own the copyrights to Supplemental Material that may be linked to, or accessed through, an article. The authors have granted ASM a non-exclusive, world-wide license to publish the Supplemental Material files. Please contact the corresponding author directly for reuse.
