# Supplementary figures and images for: Identification and characterization of a novel major facilitator superfamily (MFS) efflux pump conferring multidrug resistance in Staphylococcus aureus and Staphylococcus epidermidis
Source: Antimicrob Agents Chemother. 2025 Apr 7;69(5):e01739-24. doi: 10.1128/aac.01739-24 (PMC12057375; doi:10.1128/aac.01739-24)

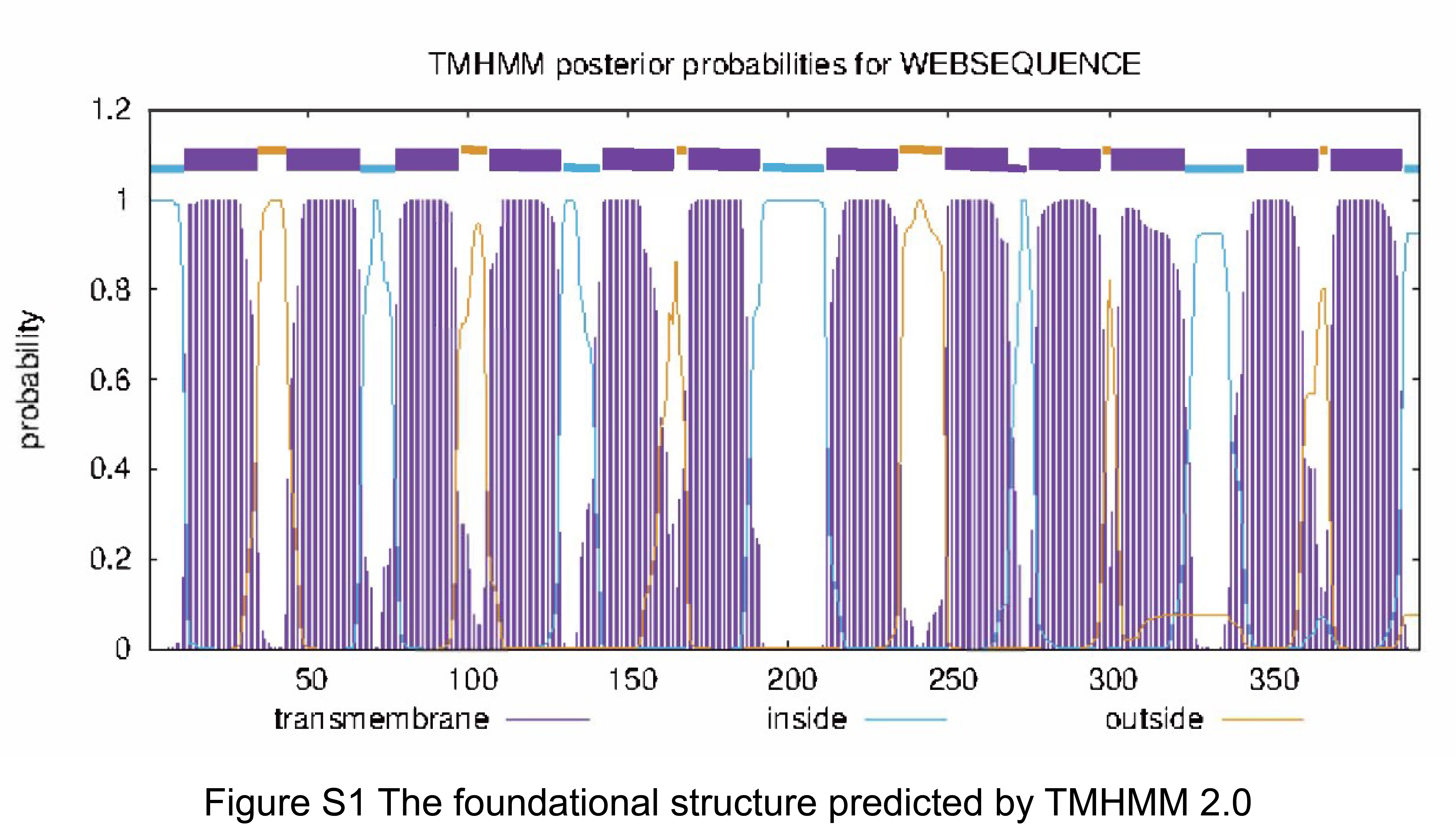

Supplement: Fig. S1 — Foundational structure predicted by TMHMM 2.0. [file aac.01739-24-s0001.jpg]

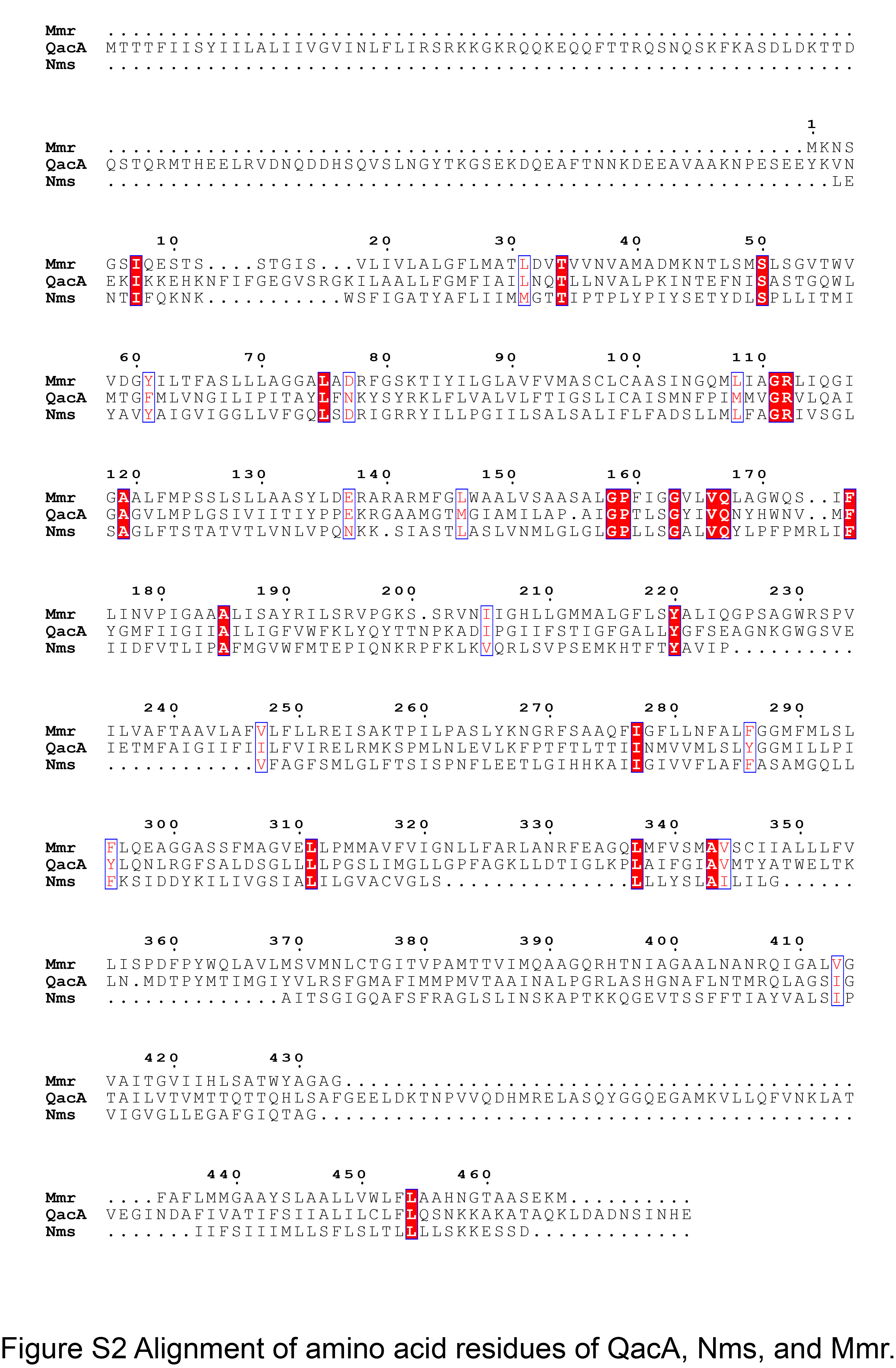

Supplement: Fig. S2 — Alignment of amino acid residues of QacA, Nms, and Mmr. [file aac.01739-24-s0002.jpg]
