# Supplementary material for: Identification and characterization of a novel major facilitator superfamily (MFS) efflux pump conferring multidrug resistance in Staphylococcus aureus and Staphylococcus epidermidis
Source: Antimicrob Agents Chemother. 2025 Apr 7;69(5):e01739-24. doi: 10.1128/aac.01739-24 (PMC12057375; doi:10.1128/aac.01739-24)
Supplement: Table S3 — Strains and plasmids used in the study. [file aac.01739-24-s0005.docx]

**Table S3 Strains and plasmids used in the study.**

| **Strains or Plasmids** | **Descriptions** | **Source** |
| --- | --- | --- |
| *E. coli* strains |  |  |
| DH5α | Recipient strains of construction of plasmids | This study |
|  |  |  |
| *S. aureus* strains |  |  |
| RN4220 | Engineering *S. aureus* strain | This study |
| RN4220+pLI50 | RN4220 transformant with the pLI50 | This study |
| RN4220+pLI50-Nms | RN4220 transformant with the pLI50 yield *nms* gene | This study |
|  |  |  |
| Plasmids |  |  |
| pLI50 | Expression vector in *S. aureus* | [22] |
| pLI50-Nms | pLI50 yield *nms* gene | This study |
